# Population genomics reveal rapid genetic differentiation in a recently invasive population of *Rattus norvegicus*

**DOI:** 10.1186/s12983-021-00387-z

**Published:** 2021-01-26

**Authors:** Yi Chen, Lei Zhao, Huajing Teng, Chengmin Shi, Quansheng Liu, Jianxu Zhang, Yaohua Zhang

**Affiliations:** 1 The State Key Laboratory of Integrated Management of Pest Insects and Rodents, Institute of Zoology, Chinese Academy of Sciences, Beijing, China; 2 CAS Center for Excellence in Biotic Interactions, University of Chinese Academy of Sciences, Beijing, China; 3 Beijing Institutes of Life Science, Chinese Academy of Sciences, Beijing, China; 4 CAS Key Laboratory of Genomic and Precision Medicine, Beijing Institute of Genomics, Chinese Academy of Sciences, Beijing, China; 5 Guangdong Key Laboratory of Animal Conservation and Resource Utilization, Guangdong Public Laboratory of Wild Animal Conservation and Utilization, Institute of Zoology, Guangdong Academy of Sciences, Guangzhou, China

**Keywords:** Population genomics, Biological invasion, Demographic history, Rapid differentiation, Ancestral range, Founder effect, *Rattus norvegicus*

## Abstract

**Background:**

Invasive species bring a serious effect on local biodiversity, ecosystems, and even human health and safety. Although the genetic signatures of historical range expansions have been explored in an array of species, the genetic consequences of contemporary range expansions have received little attention, especially in mammal species. In this study, we used whole-genome sequencing to explore the rapid genetic change and introduction history of a newly invasive brown rat (*Rattus norvegicus*) population which invaded Xinjiang Province, China in the late 1970s.

**Results:**

Bayesian clustering analysis, principal components analysis, and phylogenetic analysis all showed clear genetic differentiation between newly introduced and native rat populations. Reduced genetic diversity and high linkage disequilibrium suggested a severe population bottleneck in this colonization event. Results of TreeMix analyses revealed that the introduced rats were derived from an adjacent population in geographic region (Northwest China). Demographic analysis indicated that a severe bottleneck occurred in XJ population after the split off from the source population, and the divergence of XJ population might have started before the invasion of XJ. Moreover, we detected 42 protein-coding genes with allele frequency shifts throughout the genome for XJ rats and they were mainly associated with lipid metabolism and immunity, which could be seen as a prelude to future selection analyses in the novel environment of XJ.

**Conclusions:**

This study presents the first genomic evidence on genetic differentiation which developed rapidly, and deepens the understanding of invasion history and evolutionary processes of this newly introduced rat population. This would add to our understanding of how invasive species become established and aid strategies aimed at the management of this notorious pest that have spread around the world with humans.

**Supplementary Information:**

The online version contains supplementary material available at 10.1186/s12983-021-00387-z.

## Background

Invasive species are not only a major threat for native biodiversity (such as species decline or extinction) and ecosystems [1, 2], but also cause considerable annual damage to agriculture, property, human health and safety, and natural resources [3, 4]. It is vital to understand the dynamics of invasion processes, such as the rapid evolution and introduction history of these species, as well as their dispersal mechanisms [5–10]. Clarifying the likely spread of invasive pest is crucial for accurate risk assessment and for optimized management strategies [11]. Genomic approaches can help in developing this understanding and promise to provide higher resolution than previous genetic studies to explore the population structure, demographic history, and molecular evolution of invasive populations [12–15].

The brown rat (*Rattus norvegicus*) is one of the most successful mammalian invaders due to its remarkable migration and adaptation abilities [16, 17]. The species is believed to have originated in either northern Asia [18, 19] or Southeast Asia [20, 21], and emerged ∼1.3 million years ago [20]. Now, it has invaded and spread to nearly every major landmass except Antarctica [17, 22]. In China, the brown rat is also widespread except Tibet [16, 23, 24]. However, based on historical records, the brown rat was not observed in Xinjiang Province (XJ) until the late 1970s [25–27]. In the middle of the 1970s, brown rats were first detected on trains from Beijing to XJ, which was opened in the middle of the 1960s, and a few years later, they were first observed on land in the eastern XJ, the Turpan-Hami Basin [25, 27]. Thus, the introduction of XJ brown rats is generally attributed to Beijing-XJ railway transportation [25–27]. Despite its recent arrival, it has spread throughout XJ and there is now a large population in the region [26, 28, 29].

To further deepen the understanding of invasion history and evolutionary processes of this recently introduced rat population, we used next-generation sequencing data to explore the extent of whole-genome variation among the introduced XJ population and other native populations of brown rats in China. We explored the molecular phylogenetic relationships of the recently invasive brown rats within a broader phylogenetic framework, and tested whether introduced rats undergo rapid molecular differentiation in this new geographical range during such short period of invasion. We also investigated the source of origin and the demographic history of the introduced XJ population and identified genes with allele frequency shifts throughout the genome for XJ rats that might respond to local selection in this new geographical range.

## Results

**Fig. 1:**
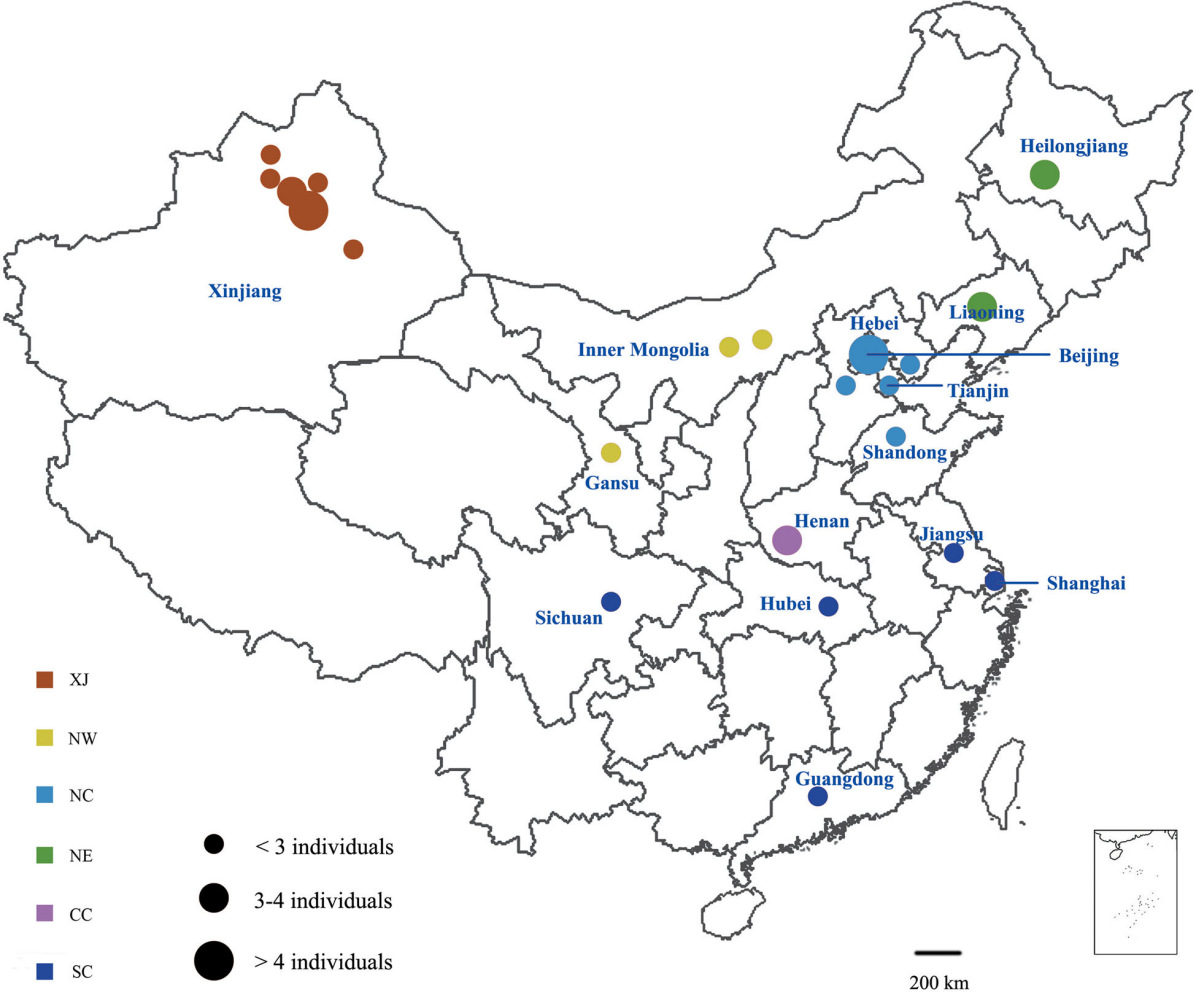
Geographic locations of the sampled rats. XJ: Xinjiang Province; NW: Northwest China; NC: North China; NE: Northeast China; CC: Central China; SC: South China

In the present study, we sequenced and analyzed the whole genomes of 50 brown rats from one invasive region and other native regions across China to an average sequencing depth of ~ 16.5× (Fig. 1; Additional file 1: Table S1). After applying stringent quality control criteria, we identified a total of 11.3 million single nucleotide polymorphisms (SNPs) among all the individuals.

### Population structure and genetic relationships among rat populations

**Fig. 2:**
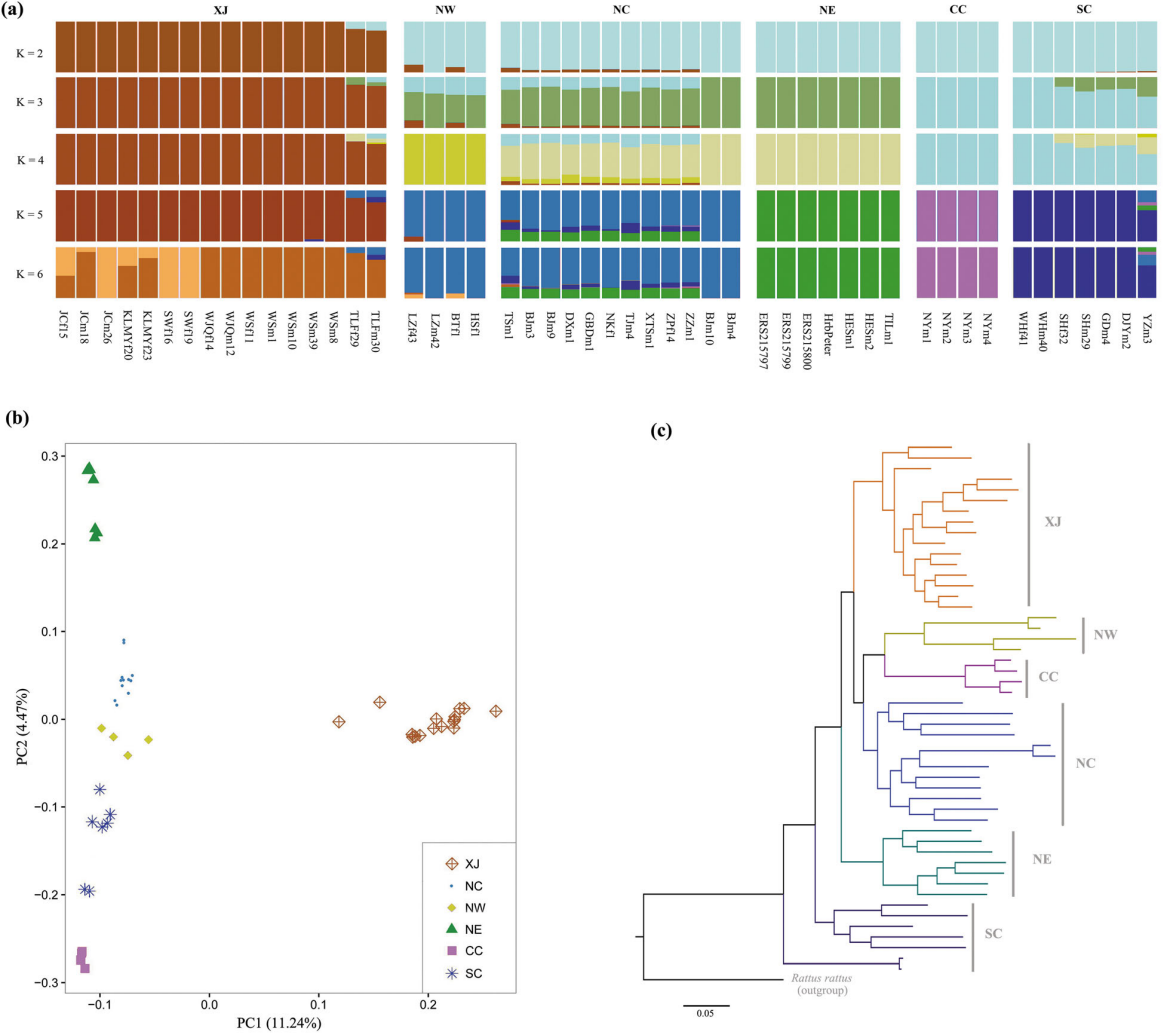
Population structure and genetic relationships among brown rat populations. **a** Genetic structure of the 50 individuals from the introduced and native populations with ADMIXTURE. The colours in each column represent the ancestry proportion, with presumed group sizes from K = 2 to K = 6. **b** Scatter plot of principal component 1 versus principal component 2 (PC1 vs. PC2) for all populations. **c** Phylogenetic tree with *Rattus rattus* as an outgroup. Group IDs correspond to those in Fig. 1

To identify population structure and the genetic relationships of different rat populations, we performed a series of classical analyses including Bayesian clustering analysis by ADMIXTURE, principal components analysis (PCA), and phylogenetic assessments using whole-genome SNPs (Fig. 2).

Clustering analyses by ADMIXTURE showed that brown rats were separated into XJ and non-XJ populations when the number of presumed ancestral populations (K) was set to 2 which is the best supported number of clusters (Fig. 2a; Additional file 1: Table S2). PCA showed that the introduced XJ rats were widely divergent from the native ones. XJ individuals clustered together in a single area and the first principal component (PC1) clearly separated XJ from other populations (NW, NE, NC, CC, and SC), which are differentiated into separate clusters along PC2 (Fig. 2b). Phylogenetic reconstruction also identified six major clusters which is consistent with both the results of the clustering analysis and the PCA (Fig. 2c). All the population-level nodes in this phylogenetic tree had a bootstrap support of 100%.

### Genetic differentiation and genetic diversity

**Fig. 3:**
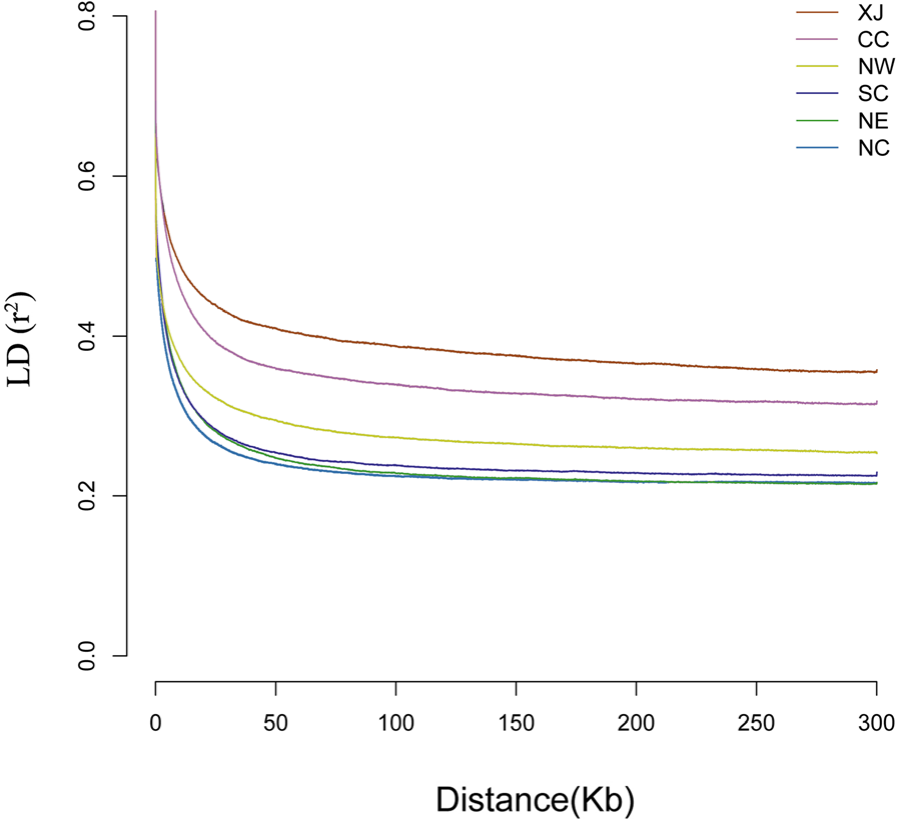
LD decay of each population. The same numbers of individuals were chosen randomly for each population to calculate r^2^. Group IDs correspond to those in Fig. 1

The genome-wide analysis of *F*_ST_ divergence showed that *F*_ST_ among groups with XJ population were much higher than those with other populations (all *P* < 2.2E− 16, Wilcoxon rank-sum test; Table 1), which further indicates the striking genetic differentiation between XJ rats and the native ones. Nucleotide diversity (π) and linkage disequilibrium (LD) were used to assess the genetic diversity within rat populations. The π in the introduced XJ population was lower than that in the other rat populations (all *P* < 2.2E− 16, Wilcoxon rank-sum test; Table 2). LD patterns showed similar trends. By calculating the pairwise LD between polymorphic sites for all regions in each population, we found that LD decayed much slower in the XJ population than in other populations (Fig. 3).

**Table 1:** *F*_ST_ among six rat populations

	XJ	NW	NC	NE	CC	SC
**XJ**	–	0.162*	0.117*	0.157*	0.218*	0.137*
**NW**	0.089	–	0.061	0.093	0.144	0.073
**NC**	0.080	0.039	–	0.041	0.104	0.030
**NE**	0.084	0.044	0.034	–	0.146	0.065
**CC**	0.103	0.059	0.050	0.061	–	0.105
**SC**	0.082	0.042	0.041	0.045	0.059	–

Note: *F*_ST_ values are presented in the top right of the matrix, and standard deviation are presented in the bottom left. The asterisks indicate statistically significant higher *F*_ST_ among groups with XJ population (*P* < 2.2E−16, Wilcoxon rank-sum test)

**Table 2:** π of all rat populations

	π (×10^**−4**^)	Standard deviation
**XJ**	11.14*	6.89
**NW**	13.29	7.14
**NC**	15.08	7.72
**NE**	13.99	7.47
**CC**	11.84	6.99
**SC**	14.91	7.82

Note: The asterisk indicates statistically significant lower π of XJ population compared with other populations (*P* < 2.2E−16, Wilcoxon rank-sum test)

### Demography and admixture of XJ rats

**Fig. 4:**
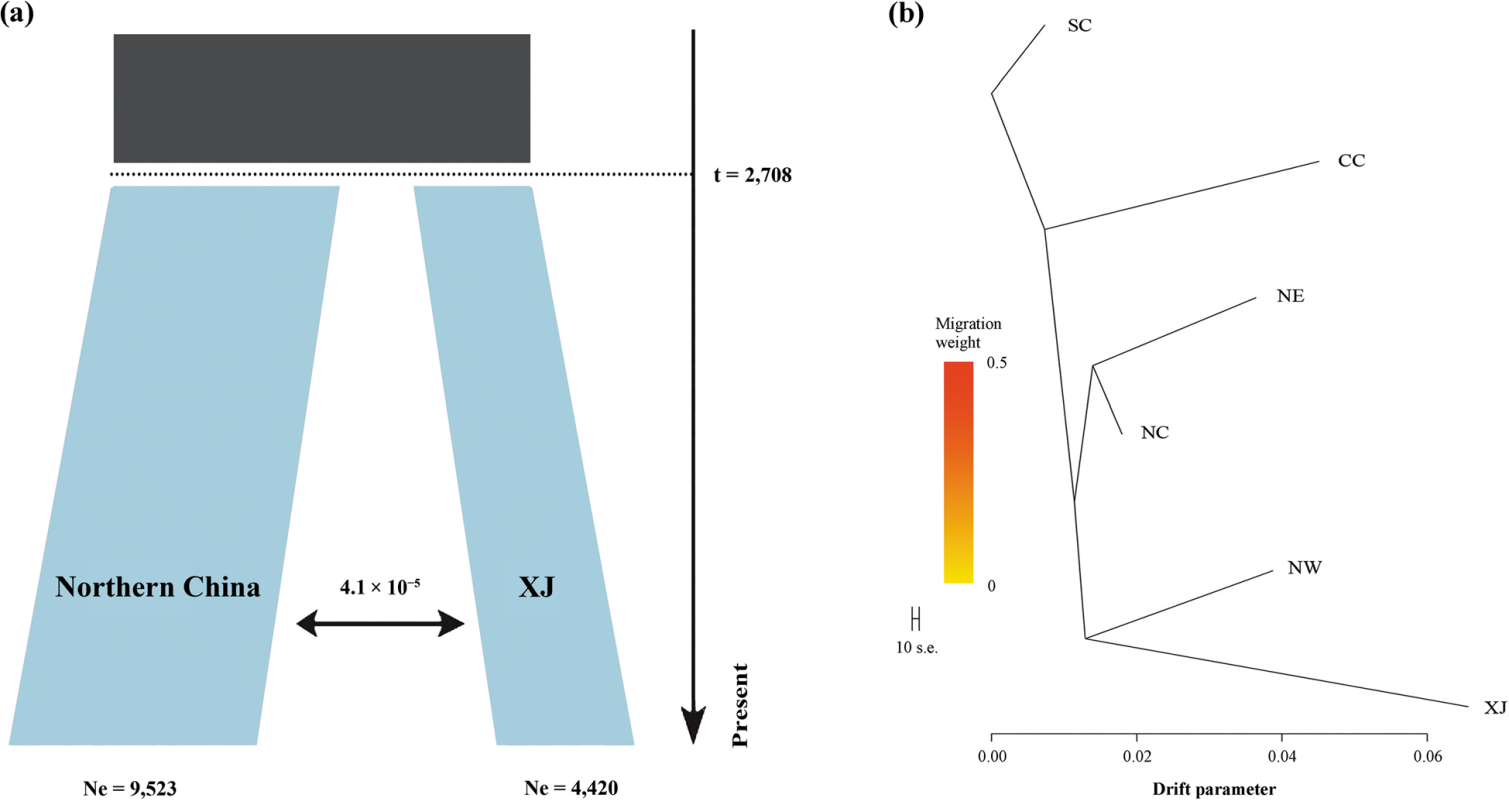
Demographic history and population relationships for XJ rats. **a** Inferred population demographic history between XJ and northern China rats using the joint site frequency spectra in δaδi [30]. Northern China included NW, NC, and NE. **b** Tree topology inferred from TreeMix [31]. Group IDs correspond to those in Fig. 1

We inferred the demographic history of the XJ population using 2D (two population) models in δ*a*δ*i* [30]. Divergence with continuous symmetric migration model yielded the best log likelihood and AIC statistic (Additional file 1: Table S3) between XJ and the northern (including NW, NC, and NE) rats. The size of the populations after the split were 4420 and 9523 for XJ and the northern rats, respectively (Fig. 4a), indicating a bottleneck of XJ population after the divergence. The divergence time between them was 2708 years (Fig. 4a), indicating that the divergence of XJ population might have started before the invasion of XJ. The estimated migration rate was quite low as 4.1 × 10^− 5^ per generation (Fig. 4a). We further tested for evidence of migration and admixture between populations using the maximum likelihood method implemented in Treemix [31]. The population tree without any migration showed that the XJ clade was most closely related to the native NW population, and these two groups formed part of a large clade of populations located in northern China, which also included the NC and NE populations (Fig. 4b), providing further evidence that the XJ rats were derived from northern China, more specifically, northwest China. There was no evidence for migration involving XJ when migration tracks were allowed in TreeMix (Additional file 2: Fig. S1). Furthermore, the F3 test showed no evidence for introgression (Additional file 2: Fig. S2). These results from Treemix were largely consistent with results from δ*a*δ*i* that indicated a very low level of introgression between XJ and other populations.

### *F*_ST_ outliers analysis

**Fig. 5:**
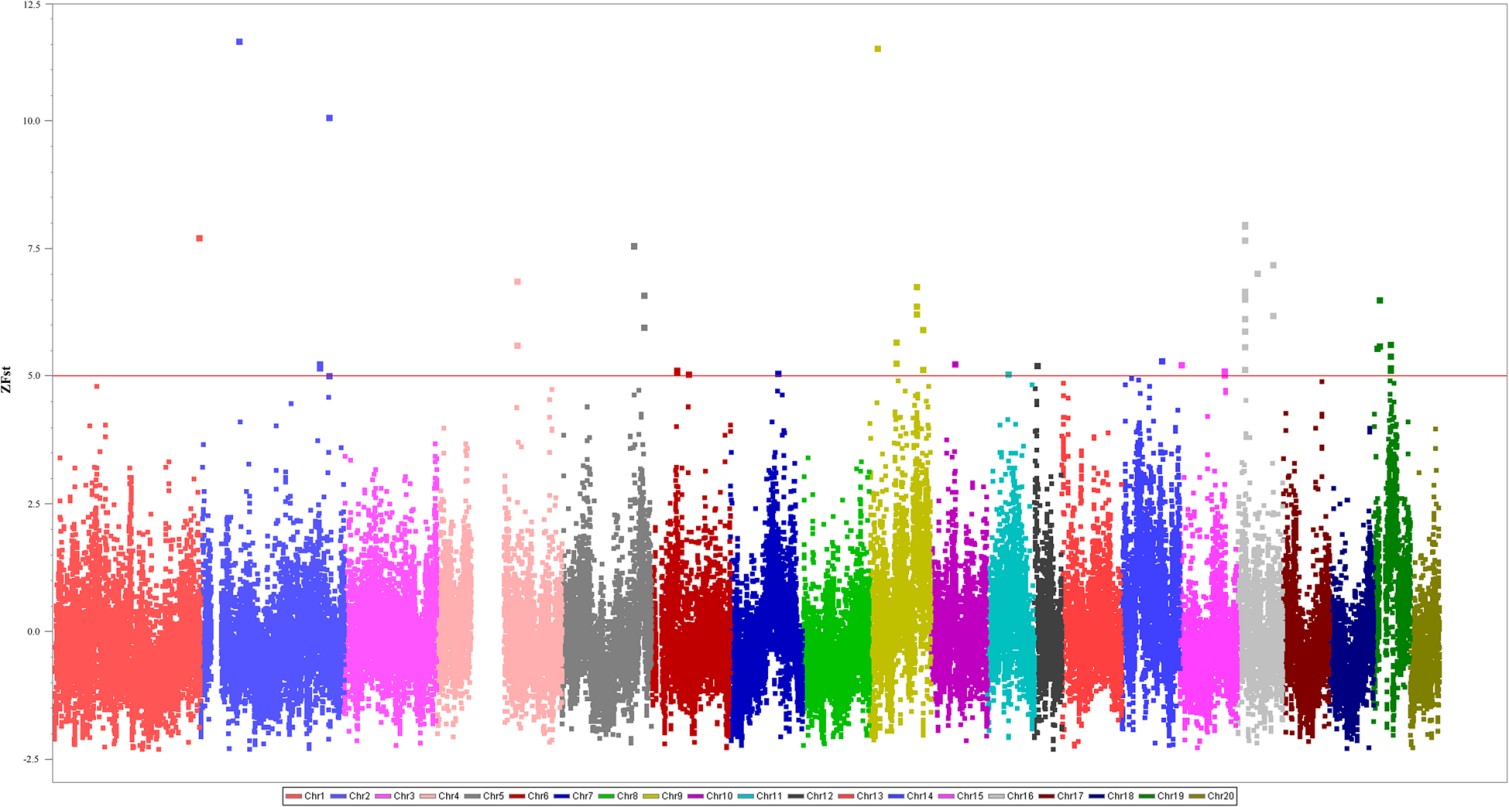
Z-transformed *F*_ST_ estimates for every 100 kb window with 50 kb steps across all chromosomes arranged from chromosome 1 to 20 (different colors). Z (*F*_ST_) illustrated for comparisons made between XJ and other native rats. Red horizontal line corresponds to 5 standard deviations from the mean

We performed a genome-wide *F*_ST_ scan to identify genes with allele-frequency shifts in XJ rats, as a prelude to future selection analyses in the novel environment of XJ. We identified 42 regions with high Z (*F*_ST_) between XJ and other native rats (Fig. 5). In these outlier regions, we detected 42 protein-coding genes (Additional file 1: Table S4) which were mainly associated with two different functions: lipid metabolism and immunity (Additional file 1: Table S5). These genes were significantly enriched in Gene Ontology (GO) terms involved in arachidonic acid secretion (GO:0050482), arachidonate transport (GO:1903963), fatty acid transport (GO:0015908), lipid catabolic process (GO:0016042), lipid transport (GO:0006869), lipase activity (GO:0016298), negative regulation of leukocyte cell-cell adhesion (GO:1903038), mannose binding (GO:0005537), monosaccharide binding (GO:0048029), etc. They also showed an overrepresentation in the Kyoto Encyclopedia of Genes and Genomes (KEGG) pathways related to linoleic acid metabolism (rno00591), fat digestion and absorption (rno04975), arachidonic acid metabolism (rno00590), C-type lectin receptor signaling pathway (rno04625), measles (rno05162), phagosome (rno04145), etc.

## Discussion

In mammals, analyses of genetic differentiation and molecular evolution among species or populations of the same species by genomic approaches have mostly focused on geologic time scales (more than tens of thousands of years) [24, 32–34]. However, rapid genetic change often occurs over ecological time scales (e.g., tens of generations or fewer) [35, 36], and multiple studies on non-mammal species have shown that evolutionary changes can occur within dozens of generations [37–42]. Our current work presents the first genomic results on rapid genetic differentiation of the recently introduced invasive brown rat. By analyzing whole-genome sequences of 50 individuals from newly introduced and native populations, we determined the phylogenetic placement of the invasive population. Our results demonstrated that the introduced XJ rats have become a recently diverged population over the past decades of invasion. All the introduced rats formed a single well-supported clade in the phylogenetic tree and showed strong genome-wide divergence in genetic structure from native ones in PCA and ADMIXTURE analyses. Such striking genetic differentiation between frontier and native rat populations implies that the invasive population of the brown rat has undergone a rapid genetic change, indicating that genetic differentiation can develop rapidly in brown rats.

With the low nucleotide diversity (π), the effective population of XJ might undergo a more recent reduction than other native ones [43]. This result is consistent with the high linkage disequilibrium (LD), suggesting that the introduced XJ rat might come from a small size of founder population and this colonization event was associated with a severe bottleneck [44]. Demographic analysis revealed that XJ population isolated from the source population ~ 2700 years ago that is much earlier than the invasion of XJ, indicating the divergence of XJ population started before the invasion of XJ. Future studies should broaden the geographical scale in northern China (especially northwest China) [45] to perform a much more accurate and comprehensive evaluation of the source population of XJ rats. Invasion and post-establishment expansions are often associated with recurrent founder effects and bottlenecks, which points to low genetic diversity and the accumulation of deleterious alleles or mutations [6, 46–49]. However, invasive populations with reduced genetic diversity may also successfully colonize new environments and expand across wide geographic area [10, 50–52]. It is noted that bottlenecks may not necessarily be negative, but help purge deleterious alleles due to genetic drift and non-random mating in small populations [53]. Nevertheless, a loss of genetic variation may limit mutations available for natural selection and is expected to impact negatively on the adaptive capacity of populations [12, 47]. The range expansion of brown rats was a response to relatively recent increases in global trade [17], although the brown rat has been widespread throughout the world. Additionally, a severe bottleneck occurred about 20 kya in the brown rat [24]. Thus the brown rat may possess the capability of rapid expansion with low effective population size.

Previous studies revealed that geography and environmental heterogeneity shaped genetic structure of brown rats [17, 54]. We also demonstrated that *R. norvegicus* was differentiated into clades (NW, NC, NE, CC, and SC) corresponding mostly to geographic locations in China. Tree topology inferred from TreeMix showed that the introduced XJ population was closely related to the native NW population, indicating that the introduced rats were derived from NW, a geographic region neighboring to XJ. In this study, our sampling range covered main distribution areas of all the four subspecies of brown rats in China [16, 23]. Tibet province that is large plateau area neighboring to XJ has no distribution of the brown rat [16, 23]. Although some other samples were also provided in the research of Zeng et al. [21], most of these sample areas were located in South China, and brown rats from these areas (including our sampling area of SC) cluster together and significantly divergent from North ones [21]. Given the large genetic distance of SC and XJ population in our current work, we believe that these unsampled areas in South China could not be the source of XJ population.

In a population with such a recent bottleneck and expansion, disentangling positive selection from neutral forces is challenging. However, we identified genes throughout the genome with significant allele frequency shifts in XJ rats, as a prelude to future selection analyses in the novel environment of XJ; A total of 42 genes were detected in *F*_ST_ outlier regions and they were mainly associated with lipid metabolism and immunity. One unique climatic characteristics of XJ is the dramatic diurnal and seasonal temperature differences [55]. The variation of lipid metabolism genes may regulate the energy metabolism of brown rats [56], consequently, fitting in with drastic changes in temperature. Rapid evolution in immune genes is a well-known example and presumably occurs because new mutations help organisms to prevail in evolutionary “arms races” with pathogens. Previous studies showed that immune-related genes were under positive selection in population divergence of the three-spined stickleback (*Gasterosteus aculeatus*) [57] or the Olympia oyster (*Ostrea lurida*) [58], indicating that how to deal with new types of pathogens might be one of the critical issues during range expansion. As the source of a variety of pathogens, immunity plays a great role in keeping health and adapting to new habitats for brown rats. Zeng et al. [21] recently demonstrated that genes related to immune system have evolved rapidly under positive selection in wild brown rats during their dispersal, indicating that resistance to external pathogens is a key issue for the brown rat in response to novel environmental forces.

## Conclusions

This work highlights that rapid genetic change can occur after such a short period of invasion in brown rats, and the successful invasion could come from a small size of founder population. Geography and environmental heterogeneity shaped genetic structure of brown rats, and the adjacent NW population was the source of XJ population. Invasive species can rapidly adapt to the changed conditions by means of genetic changes through the process of evolution [8, 59, 60]. Identifying the underlying genetic causes of invasion success is a key component of research on biological invasion [6, 61, 62]. This study presents the first genomic evidence on genetic differentiation which developed rapidly, and deepens the understanding of invasion history and evolutionary processes of this recently introduced rat population, which would hold clues to their successful invasion and could aid strategies aimed at the management of this notorious pest that have spread around the world with humans.

## Methods

### Samples, study sites, and genome sequencing

A total of 50 adult brown rats were sampled across China including Xinjiang and other 14 provinces, which cover the main distribution area of brown rats in China [16, 23] (Fig. 1; Additional file 1: Table S1). Rats were trapped with snap trap and the nearest trapping locations among collected rats were 5 km apart to avoid sampling closely related individuals. Tails were obtained and stored in ethanol for DNA extraction. The species status of brown rats was confirmed via morphology and mitochondrial cytochrome oxidase subunit I barcode sequences by Sanger sequencing [24].

Whole-genomic DNA of 38 individuals was extracted from small pieces of tail tissue using a TailGen DNA extraction kit (CWBIO, Beijing, China). The quality and integrity of the extracted DNA was checked by measuring the A260/A280 ratio using a NanoDrop ND-1000 spectrophotometer (Thermo Fisher Scientific Inc., Waltham, MA, USA) and by agarose gel electrophoresis. Library preparation, Illumina sequencing, read alignments, and variant calling were performed by Annoroad Gene Technology (Beijing) Co. Ltd. For each individual, 100 ng genomic DNA was used to construct PCR-based libraries with a 350 base pair (bp) insertion size and sequenced on an Illumina HiSeq X Ten instrument with 150 bp paired-end reads. Our previously generated 12 whole genome sequences of wild-caught brown rat individuals [24] were reanalyzed in this study. After filtering out raw sequencing reads containing adapters and reads of low quality, the remaining clean reads were mapped to the reference *R. norvegicus* genome RGSC5.0 [63] using BWA v0.7.12 with default parameters [64]. SAMtools v1.2 [65] was performed to sort reads, and MarkDuplicates in Picard tools v1.13 (http://broadinstitute.github.io/picard/) was used to remove PCR duplicates. Reads mapped to two or more locations were filtered out.

### SNP calling and filtering

The Genome Analysis Toolkit (GATK) [66] HaplotypeCaller protocol was used for SNP calling via local re-assembly of haplotypes for the populations. SNPs were further filtered by applying the following cutoffs with the GATK VariantFiltration protocol: QD < 10.0, FS > 10.0, DP < 4.0, QUAL < 30.0, ReadPosRankSum < − 8.0. Additionally, the sites with a minor allele frequency (MAF) < 0.05 or including more than 10% missing genotypes were filtered out. Then the filtered high-quality SNPs were kept for subsequent analysis.

### Population structure analyses

We used ADMIXTURE v1.3.0 [67] to investigate the population structure, with the number of coancestry clusters ranging from 2 to 6. To assess the best value of K, we performed 10-fold cross-validation and determined the K value with the lowest cross-validation error. PCA was performed using EIGENSOFT v6.0.1 [68]. To mitigate the effects of linkage disequilibrium (LD) on genetic structure, we pruned the markers using the “-indep-pairwise 50 5 0.05” option of PLINK [69]. To investigate the relationships within introduced and native populations, a phylogenetic tree from whole-genome SNP data was constructed using SNPhylo [70]. The program was run with 100 bootstrap repetitions, and the genome information of *R. rattus* [32] was used as an outgroup.

### Genetic differentiation, genetic diversity, and LD analyses

Pairwise genetic differentiation and nucleotide diversity among rat populations were calculated using VCFtools v0.1.16 [71] by means of *F*_ST_ (−weir-fst) and π (−site-pi) with a 100 kb sliding window, respectively. Linkage disequilibrium (LD) decay was calculated with PopLDdecay [72] with the following parameters: -MaxDist 300 -MAF 0.05 -Miss 0.9.

### Demographic history

To investigate alternative divergence scenarios for XJ population, we used the diffusion approximation method of δ*a*δ*i* to analyze two-dimensional joint site frequency spectra (2D-JSFS) [30]. We used the demographic modelling pipeline (dadi_pipeline) of Portik et al. [73] to conduct all analyses. For all models, we performed consecutive rounds of optimizations following Portik et al. [73]. For each round, we ran multiple replicates and used parameter estimates from the best scoring replicate (highest log-likelihood) to seed searches in the following round. We used the default settings in dadi_pipeline for each round (replicates = 10, 20, 30, 40; maxiter = 3, 5, 10, 15; fold = 3, 2, 2, 1), and optimized parameters using the Nelder-Mead method (optimize_log_fmin). Akaike Information Criteria (AIC) values were used to compare demography models, and the demography model with the lowest AIC was chosen as the best-fitting model. We used the approach described in Mattingsdal et al. [74] and Choi et al. [75] to convert the parameter estimates into meaningful biological values using a generation time of 0.5 year and a mutation rate of 2.96 × 10^− 9^ [24, 32].

### Population admixture analysis

Population tree topology was estimated using the maximum likelihood method implemented in TreeMix [31]. TreeMix models the genetic drift at genome-wide polymorphisms to infer relationships between populations. It first estimates a dendrogram of the relationships between sampled populations. Next it compares the covariance structure modeled by this dendrogram to the observed covariance between populations. When populations are more closely related than modeled by a bifurcating tree it suggests that there has been admixture in the history of those populations. TreeMix then adds an edge to the phylogeny, now making it a phylogenetic network. The position and direction of these edges are informative; if an edge originates more basally in the phylogenetic network it indicates that this admixture occurred earlier in time or from a more diverged population. We first inferred the maximum likelihood (ML) tree with the command “-i input -o output.” We then tested trees for one and two migration events (Additional file 2: Fig. S2). The threepop module (F3 test) from the TreeMix package was used to validate the migration events.

### *F*_ST_ outliers analysis

We estimated the *F*_ST_ (XJ/others) values for each window using VCFtools v0.1.16 (Danecek et al., 2011), with a window size of 100 kb and a step size of 50 kb. Next, we Z-transformed *F*_ST_ values and then identified regions with Z (*F*_ST_) that were greater than 5 standard deviations from the mean [40, 76, 77]. To characterize the molecular functions of the genes contained in these outlier regions, we performed functional enrichment analyses using the clusterProfiler toolkit [78], where the significance level was set at 0.05 and the *P*-value was corrected using the Benjamini-Hochberg false discovery rate (FDR).

## Supplementary Information

**Additional file 1: Table S1.** Sample information and sequencing statistics. **Table S2.** Cross-validation errors for different K values. **Table S3.** Best replicate of each of the optimized demographic models using ∂a∂i. **Table S4.** 42 genes in outlier regions between XJ and other native populations. **Table S5.** Functional enrichment of genes with allele frequency shifts throughout the genome for XJ rats.

**Additional file 2: Figure S1.** Tree topology inferred from TreeMix with m = 1 (a) and m = 2 (b). Group IDs correspond to those in Fig. 1. **Figure S2.** Detection of genetic mixture across all subgroups. Significance of 3 Population Test (Z) represents whether the corresponding subgroup (on Y axis) is of mixed ancestry of other subgroups. Each dot indicates the Z score of a test between the target subgroup and every pair of other subgroups. Positive value suggests a result of unadmixed. All the groups were showed with only positive values, suggesting a relatively unadmixed relationship to other subgroups. Group IDs correspond to those in Fig. 1.

## Data Availability

The genome sequencing data are available on the Genome Sequence Archive database (http://gsa.big.ac.cn/) under accession numbers CRA001635.

## Abbreviations

XJ: Xinjiang Province
NW: Northwest China
NC: North China
NE: Northeast China
CC: Central China
SC: South China
SNP: Single nucleotide polymorphism
PC: Principal component
PCA: Principal components analysis
LD: Linkage disequilibrium
