# Supplementary material for: Population genomics reveal rapid genetic differentiation in a recently invasive population of Rattus norvegicus
Source: Front Zool. 2021 Jan 26;18:6. doi: 10.1186/s12983-021-00387-z (PMC7836188; doi:10.1186/s12983-021-00387-z)
Supplement: Supplementary file 1 — Additional file 1: Table S1. Sample information and sequencing statistics. Table S2. Cross-validation errors for different K values. Table S3. Best replicate of each of the optimized demographic models using ∂a∂i. Table S4. 42 genes in outlier regions between XJ and other native populations. Table S5. Functional enrichment of genes with allele frequency shifts throughout the genome for XJ rats. [file 12983_2021_387_MOESM1_ESM.docx]

**Table S1**. Sample information and sequencing statistics.

| **Sample** | **Region** | **Locality** | **Sex** | **Clean Reads** | **Mapped Bases** | **Mapping Rate (%)** | **Mean Depth** | **Reference Genome**  **Coverage (>1×) (%)** |
| --- | --- | --- | --- | --- | --- | --- | --- | --- |
| KLMYf20^*^ | XJ | Kelamayi, Xinjiang Uygur Autonomous Region, P.R.China | F | 218,295,056 | 31,844,567,591 | 97.25 | 12.22 | 98.77 |
| KLMYf23^*^ | XJ | Kelamayi, Xinjiang Uygur Autonomous Region, P.R.China | F | 239,532,950 | 34,973,346,939 | 97.34 | 13.37 | 98.92 |
| SWf16^*^ | XJ | Shawan, Xinjiang Uygur Autonomous Region, P.R.China | F | 284,874,092 | 41,595,749,758 | 97.34 | 15.93 | 99.07 |
| SWf19^*^ | XJ | Shawan, Xinjiang Uygur Autonomous Region, P.R.China | F | 260,198,392 | 38,037,791,869 | 97.46 | 14.57 | 99.02 |
| JCf15^*^ | XJ | Changji, Xinjiang Uygur Autonomous Region, P.R.China | F | 304,283,712 | 44,462,792,009 | 97.42 | 17.00 | 99.13 |
| JCm18^*^ | XJ | Changji, Xinjiang Uygur Autonomous Region, P.R.China | M | 257,888,888 | 37,575,639,170 | 97.14 | 14.44 | 98.94 |
| JCm26^*^ | XJ | Changji, Xinjiang Uygur Autonomous Region, P.R.China | M | 259,039,128 | 37,681,989,857 | 96.98 | 14.51 | 99 |
| WSf11^*^ | XJ | Urumqi, Xinjiang Uygur Autonomous Region, P.R.China | F | 225,021,402 | 32,867,298,900 | 97.38 | 12.60 | 98.85 |
| WSm10^*^ | XJ | Urumqi, Xinjiang Uygur Autonomous Region, P.R.China | M | 338,243,022 | 49,347,132,674 | 97.26 | 18.90 | 99.23 |
| WSm1^*^ | XJ | Urumqi, Xinjiang Uygur Autonomous Region, P.R.China | M | 277,130,762 | 40,414,670,744 | 97.22 | 15.40 | 99.05 |
| WSm39^*^ | XJ | Urumqi, Xinjiang Uygur Autonomous Region, P.R.China | M | 290,631,222 | 42,353,178,334 | 97.15 | 16.25 | 99.11 |
| WSm8^*^ | XJ | Urumqi, Xinjiang Uygur Autonomous Region, P.R.China | M | 294,012,276 | 42,846,861,525 | 97.15 | 16.44 | 99.07 |
| WJQf14^*^ | XJ | Wujiaqu, Xinjiang Uygur Autonomous Region, P.R.China | F | 259,318,340 | 37,862,797,304 | 97.34 | 14.50 | 99 |
| WJQm12^*^ | XJ | Wujiaqu, Xinjiang Uygur Autonomous Region, P.R.China | M | 297,868,198 | 43,438,302,627 | 97.22 | 16.66 | 99.22 |
| TLFf29^*^ | XJ | Tulufan, Xinjiang Uygur Autonomous Region, P.R.China | F | 297,353,340 | 43,424,875,546 | 97.36 | 16.62 | 99.05 |
| TLFm30^*^ | XJ | Tulufan, Xinjiang Uygur Autonomous Region, P.R.China | M | 241,902,740 | 35,049,362,660 | 96.59 | 13.37 | 98.99 |
| LZf43^*^ | NW | Lanzhou, Gansu Province, P.R.China | F | 228,569,396 | 33,185,220,018 | 96.79 | 12.73 | 99.05 |
| LZm42^*^ | NW | Lanzhou, Gansu Province, P.R.China | M | 318,747,356 | 46,203,560,108 | 96.64 | 17.72 | 99.19 |
| BTf1^*^ | NW | Baotou, Inner Mongolia Autonomous Region, P.R.China | F | 253,359,024 | 36,731,807,228 | 96.65 | 14.02 | 99.11 |
| HSf1^*^ | NW | Huhhot, Inner Mongolia Autonomous Region, P.R.China | F | 231,586,072 | 33,573,544,812 | 96.65 | 12.89 | 98.77 |
| NKf1^*^ | NC | Beijing, P.R.China | F | 284,164,786 | 41,533,884,007 | 97.44 | 15.93 | 98.99 |
| XTSm1^*^ | NC | Beijing, P.R.China | M | 312,091,200 | 45,429,360,290 | 97.04 | 17.41 | 99.19 |
| BJm10^*^ | NC | Beijing, P.R.China | M | 257,879,898 | 37,299,669,050 | 96.43 | 14.35 | 99.2 |
| BJm4^*^ | NC | Beijing, P.R.China | M | 231878942 | 33567149244 | 96.51 | 12.98 | 99.04 |
| BJm9^*^ | NC | Beijing, P.R.China | M | 219112308 | 31704106860 | 96.46 | 12.23 | 99.1 |
| BJm3 | NC | Beijing, P.R.China | M | 389281544 | 47076576717 | 93.85 | 16.18 | 98.82 |
| DXm1^*^ | NC | Beijing, P.R.China | M | 340628120 | 49251048063 | 96.39 | 18.90 | 99.34 |
| ZZm1^*^ | NC | Baoding, Hebei Province, P.R.China | M | 303624020 | 44205878953 | 97.06 | 16.97 | 99.23 |
| GBDm1^*^ | NC | Baoding, Hebei Province, P.R.China | M | 248418132 | 35960435457 | 96.51 | 13.77 | 98.97 |
| TSm1^*^ | NC | Tangshan, Hebei Province, P.R.China | M | 247159274 | 36042733850 | 97.22 | 13.68 | 99.02 |
| TJm4^*^ | NC | Tianjin, P.R.China | M | 201944142 | 29251167387 | 96.57 | 11.27 | 99.01 |
| ZPf14^*^ | NC | Binzhou, Shandong Province, P.R.China | F | 248320530 | 36251864485 | 97.33 | 13.92 | 99.03 |
| ERS215797 | NE | Harbin, Heilongjiang Province, P.R.China | M | 717078215 | 70725385817 | 99.84 | 24.31 | 98.92 |
| ERS215799 | NE | Harbin, Heilongjiang Province, P.R.China | F | 630470497 | 62174922846 | 99.83 | 21.37 | 98.86 |
| ERS215800 | NE | Harbin, Heilongjiang Province, P.R.China | F | 709893323 | 69594922683 | 99.23 | 23.92 | 98.93 |
| HrbPeter | NE | Harbin, Heilongjiang Province, P.R.China | F | 1267503343 | 1.20199E+11 | 95.34 | 41.31 | 99.06 |
| HESm1^*^ | NE | Jinzhou, Liaoning Province, P.R.China | M | 212658846 | 30672287146 | 96.15 | 11.80 | 99 |
| HESm2^*^ | NE | Jinzhou, Liaoning Province, P.R.China | M | 203335760 | 29365607346 | 96.28 | 11.32 | 98.9 |
| TILm1^*^ | NE | Tieling, Liaoning Province, P.R.China | M | 224114170 | 32489342539 | 96.65 | 12.54 | 98.94 |
| NYm1 | CC | Nanyang, Henan Province, P.R.China | M | 576323865 | 71642774669 | 93.29 | 24.62 | 98.94 |
| NYm2 | CC | Nanyang, Henan Province, P.R.China | M | 491309325 | 59955244299 | 93.91 | 20.61 | 98.95 |
| NYm3 | CC | Nanyang, Henan Province, P.R.China | M | 581926414 | 72480146793 | 93.67 | 24.91 | 98.99 |
| NYm4 | CC | Nanyang, Henan Province, P.R.China | M | 448033201 | 53587335205 | 94.25 | 18.42 | 98.86 |
| WHf41^*^ | SC | Wuhan, Hubei Province, P.R.China | F | 265731964 | 38578805736 | 96.79 | 14.82 | 99.18 |
| WHm40^*^ | SC | Wuhan, Hubei Province, P.R.China | M | 249640704 | 36162123526 | 96.57 | 13.88 | 99.04 |
| GDm4 | SC | Guangzhou, Guangdong Province, P.R.China | M | 527739986 | 65433672685 | 93.50 | 22.49 | 98.98 |
| SHf32^*^ | SC | Shanghai, P.R.China | F | 241159198 | 34952172529 | 96.62 | 13.41 | 98.98 |
| SHm29^*^ | SC | Shanghai, P.R.China | M | 291476308 | 42235856609 | 96.60 | 16.19 | 99.24 |
| DJYm2 | SC | Chengdu, Sichuan Province, P.R.China | M | 257803807 | 28129230864 | 94.08 | 9.67 | 98.58 |
| YZm3 | SC | Yangzhou, Jiangsu Province, P.R.China | M | 451243189 | 55953666509 | 93.06 | 19.23 | 98.93 |

The asterisk means that the sample was newly collected, while others were relied on our previous existing data. XJ: Xinjiang Province; NW: Northwest China; NC: North China; NE: Northeast China; CC: Central China; SC: South China.

**Table S2**. Cross-validation errors for different K values.

| K | Cross-validation error |
| --- | --- |
| 1 | 0.62908 |
| 2 | 0.59544 |
| 3 | 0.61272 |
| 4 | 0.66764 |
| 5 | 0.72330 |
| 6 | 0.73717 |

**Table S3.** Best replicate of each of the optimized demographic models using ∂a∂i.

| **Model** | **Replicate** | **log-likelihood** | **AIC** | **chi-squared** | **theta** | **optimized_params** |
| --- | --- | --- | --- | --- | --- | --- |
| sym_mig | Round_4_Replicate_10 | -14690.3 | 29388.54 | 27365.17 | 52240.06 | 0.0879,0.0408,19.0069,0.025 |
| sym_mig_size | Round_4_Replicate_36 | -17903.5 | 35820.9 | 31929.49 | 13580.51 | 16.4976,1.9934,0.5302,0.2932,2.723,4.9524,0.32 |
| sec_contact_sym_mig | Round_4_Replicate_5 | -20050.3 | 40110.54 | 41297.33 | 9084.87 | 0.5041,0.2561,3.3104,27.247,0.9236 |
| sec_contact_sym_mig_size | Round_4_Replicate_7 | -20254.8 | 40523.5 | 40923.67 | 13925.75 | 0.8937,0.5475,0.4132,0.2068,3.9722,15.3033,0.7241 |
| vic_sec_contact_sym_mig | Round_4_Replicate_17 | -22457.5 | 44922.9 | 43680.68 | 14052.2 | 2.5369,9.8854,0.8964,0.332 |
| asym_mig | Round_4_Replicate_33 | -22889.8 | 45789.6 | 42047.01 | 46892.68 | 0.2047,0.1098,8.5216,4.7524,0.0634 |
| asym_mig_twoepoch | Round_4_Replicate_37 | -23325.6 | 46667.18 | 40686.59 | 71444.17 | 0.2177,0.0621,0.0331,0.1818,5.5575,15.9157,0.1116,0.1877 |
| sec_contact_asym_mig | Round_4_Replicate_22 | -23410.2 | 46832.38 | 46513.46 | 6058.34 | 1.3,0.6935,0.7144,1.4209,24.7514,1.5634 |
| no_mig | Round_4_Replicate_38 | -25640.8 | 51287.52 | 51974.01 | 43454.79 | 0.0924,0.0464,0.0119 |
| sec_contact_asym_mig_size | Round_4_Replicate_10 | -25794.6 | 51605.16 | 50989.81 | 6447.82 | 0.1456,0.7707,3.0199,0.3573,0.8466,3.0946,19.8669,0.8825 |
| founder_asym | Round_4_Replicate_16 | -26705.4 | 53420.76 | 44820.08 | 41247.11 | 0.0598,2.9741,13.925,0.1575,0.4825 |
| sym_mig_twoepoch | Round_4_Replicate_15 | -29329.3 | 58670.62 | 52912.49 | 22924.55 | 0.6789,0.3225,0.0943,2.4645,3.9502,0.6238 |
| anc_asym_mig_size | Round_4_Replicate_21 | -30284.9 | 60585.78 | 50299.34 | 40961.6 | 0.1975,0.0594,28.6858,1.6612,0.1155,15.6095,0.0323,0.01 |
| founder_nomig_admix_late | Round_4_Replicate_14 | -30432.2 | 60872.32 | 52597.45 | 37240.45 | 0.0484,0.0469,0.4393,0.0839 |
| founder_sym | Round_4_Replicate_33 | -31433.5 | 62875.08 | 57075.45 | 38696.64 | 0.1025,4.8,0.1269,0.4966 |
| founder_nomig_admix_two_epoch | Round_4_Replicate_14 | -33041 | 66091.92 | 58157.49 | 37452.64 | 0.1103,0.1172,0.0101,0.4246,0.3469 |
| vic_sec_contact_asym_mig | Round_4_Replicate_16 | -33751.2 | 67512.42 | 56191.93 | 30530.45 | 0.9974,6.3994,1.1399,0.4098,0.174 |
| asym_mig_size | Round_4_Replicate_9 | -35683 | 71381.98 | 59234.42 | 22874.52 | 2.103,0.4951,1.3437,0.1485,0.7125,7.4247,0.1788,14.8915 |
| anc_sym_mig_size | Round_4_Replicate_26 | -35887.8 | 71789.64 | 61433.07 | 15887.55 | 2.1608,0.6571,0.1041,0.5636,1.8308,9.0669,0.0107 |
| vic_two_epoch_admix | Round_4_Replicate_33 | -36060.2 | 72128.44 | 62632.75 | 34811.81 | 0.0841,0.0107,0.0809,0.4547 |
| founder_nomig | Round_4_Replicate_12 | -36824.6 | 73655.28 | 73309.73 | 37435.37 | 0.0362,0.0277,0.5 |
| founder_nomig_admix_early | Round_4_Replicate_20 | -36851.5 | 73710.92 | 73482.79 | 37419.67 | 0.0348,0.0267,0.4996,0.0865 |
| vic_no_mig_admix_late | Round_4_Replicate_11 | -38158.8 | 76323.52 | 66048.92 | 35839.14 | 0.0202,0.0501,0.0667 |
| anc_sym_mig | Round_4_Replicate_6 | -39812.9 | 79635.7 | 74898.35 | 36846.14 | 0.4186,0.2369,2.0422,0.0538,0.0125 |
| anc_asym_mig | Round_4_Replicate_30 | -42961.7 | 85935.38 | 74726.98 | 9710.34 | 2.1657,1.1153,0.724,1.2789,12.9222,0.0443 |
| vic_anc_sym_mig | Round_4_Replicate_3 | -43856.6 | 87721.16 | 76889.21 | 32147.84 | 2.8198,1.6393,0.0101,0.3697 |
| vic_no_mig | Round_4_Replicate_17 | -44217.8 | 88439.54 | 89130.82 | 36391.86 | 0.01,0.0338 |
| vic_no_mig_admix_early | Round_4_Replicate_26 | -44218.1 | 88442.16 | 89047.57 | 36387.63 | 0.0101,0.0341,0.3147 |
| vic_anc_asym_mig | Round_4_Replicate_24 | -45232.7 | 90475.44 | 81191.14 | 35703.07 | 0.1912,9.9275,1.1393,0.0105,0.1723 |
| no_mig_size | Round_4_Replicate_13 | -45722.2 | 91456.36 | 90927.31 | 34249.48 | 16.7043,6.4209,0.5344,0.2187,0.0151,0.0493 |

These replicates were ordered as-per their log-likelihoods. The model with the highest log-likelihood was considered as the most likely model.

**Table S4.** 42 genes in outlier regions between XJ and other populations.

| **Gene symble** | **Description** | **Outlier region** | **Z*F*_ST_** |
| --- | --- | --- | --- |
| *GFRA1* | GDNF family receptor alpha 1 | Chr1: 286750001——286850001 | 7.752854 |
| *COL11A1* | collagen type XI alpha 1 chain | Chr2: 234850001——235000001 | 5.241958 |
| *DCM5* | Dcm5 protein | Chr2: 253500001——253700001 | 7.587815 |
| *ELOVL6* | ELOVL fatty acid elongase 6 | Chr2: 253500001——253700001 | 7.587815 |
| *OLR855* | olfactory receptor 855 | Chr5: 141250001——141350001 | 7.59278 |
| *OLR856* | olfactory receptor 856 | Chr5: 141250001——141350001 | 7.59278 |
| *OLR858* | olfactory receptor 858 | Chr5: 141250001——141350001 | 7.59278 |
| *PLA2G2A* | phospholipase A2 group IIA | Chr5: 160900001——161050001 | 6.318227 |
| *PLA2G2C* | phospholipase A2, group IIC | Chr5: 160900001——161050001 | 6.318227 |
| *PLA2G2D* | phospholipase A2, group IID | Chr5: 160900001——161050001 | 6.318227 |
| *PLA2G2F* | phospholipase A2, group IIF | Chr5: 160900001——161050001 | 6.318227 |
| *PLA2G5* | phospholipase A2, group V | Chr5: 160900001——161050001 | 6.318227 |
| *UBXN10* | UBX domain protein 10 | Chr5: 160900001——161050001 | 6.318227 |
| *SAMD12* | sterile alpha motif domain containing 12 | Chr7: 93850001——93950001 | 5.103451 |
| *SLCO6B1* | solute carrier organic anion transporter family member 6B1 | Chr9: 104000001——104150001 | 5.572895 |
| *ARMC9* | armadillo repeat containing 9 | Chr9: 92800001——93000001 | 6.498974 |
| *HTR2B* | 5-hydroxytryptamine receptor 2B | Chr9: 92800001——93000001 | 6.498974 |
| *PSMD1* | proteasome 26S subunit, non-ATPase 1 | Chr9: 92800001——93000001 | 6.498974 |
| *ATPAF2* | ATP synthase mitochondrial F1 complex assembly factor 2 | Chr10: 46500001——46600001 | 5.277405 |
| *DRC3* | dynein regulatory complex subunit 3 | Chr10: 46500001——46600001 | 5.277405 |
| *DRG2* | developmentally regulated GTP binding protein 2 | Chr10: 46500001——46600001 | 5.277405 |
| *GID4* | GID complex subunit 4 | Chr10: 46500001——46600001 | 5.277405 |
| *MYO15A* | myosin XVA | Chr10: 46500001——46600001 | 5.277405 |
| *BRWD1* | bromodomain and WD repeat domain containing 1 | Chr11: 39900001——40000001 | 5.091127 |
| *CCL25* | C-C motif chemokine ligand 25 | Chr12: 4500001——4600001 | 5.256742 |
| *CD209A* | CD209a molecule | Chr12: 4500001——4600001 | 5.256742 |
| *CD209B* | CD209b antigen | Chr12: 4500001——4600001 | 5.256742 |
| *CD209E* | CD209e molecule | Chr12: 4500001——4600001 | 5.256742 |
| *DUSP13* | dual specificity phosphatase 13 | Chr15: 2500001——2600001 | 5.26594 |
| *LMO7* | LIM domain 7 | Chr15: 87900001——88050001 | 5.111552 |
| *CCSER2* | coiled-coil serine-rich protein 2 | Chr16: 13400001——14000001 | 6.653484 |
| *RGD1308750* | similar to DNA segment, Chr 19, Brigham & Womens Genetics 1357 expressed | Chr16: 69700001——69850001 | 6.733033 |
| *RGD1561481* | similar to ubiquitin specific protease 12 | Chr16: 69700001——69850001 | 6.733033 |
| *VMA21* | VMA21 vacuolar H+-ATPase homolog | Chr16: 69700001——69850001 | 6.733033 |
| *ADGRG1* | adhesion G protein-coupled receptor G1 | Chr19: 10300001——10450001 | 6.086116 |
| *ADGRG3* | adhesion G protein-coupled receptor G3 | Chr19: 10300001——10450001 | 6.086116 |
| *DRC7* | dynein regulatory complex subunit 7 | Chr19: 10300001——10450001 | 6.086116 |
| *KATNB1* | katanin regulatory subunit B1 | Chr19: 10300001——10450001 | 6.086116 |
| *KIFC3* | kinesin family member C3 | Chr19: 10300001——10450001 | 6.086116 |
| *ABCC12* | ATP binding cassette subfamily C member 12 | Chr19: 32350001——32600001 | 5.366233 |
| *LONP2* | lon peptidase 2, peroxisomal | Chr19: 32350001——32600001 | 5.366233 |
| *SIAH1* | siah E3 ubiquitin protein ligase 1 | Chr19: 32350001——32600001 | 5.366233 |

**Table S5.** Functional enrichment of genes with allele frequency shifts throughout the genome for XJ rats.

| **ID** | **Terms** | **Class** | **Gene number** | ***P* value** | **Corrected *P* value** |
| --- | --- | --- | --- | --- | --- |
| GO:0050482 | arachidonic acid secretion | BP | 5 | 4.11E-09 | 1.15E-06 |
| GO:1903963 | arachidonate transport | BP | 5 | 4.11E-09 | 1.15E-06 |
| GO:0032309 | icosanoid secretion | BP | 5 | 3.58E-08 | 6.55E-06 |
| GO:0071715 | icosanoid transport | BP | 5 | 5.84E-08 | 6.55E-06 |
| GO:1901571 | fatty acid derivative transport | BP | 5 | 5.84E-08 | 6.55E-06 |
| GO:0015909 | long-chain fatty acid transport | BP | 5 | 1.26E-07 | 1.18E-05 |
| GO:0015908 | fatty acid transport | BP | 5 | 8.75E-07 | 7.01E-05 |
| GO:0046717 | acid secretion | BP | 5 | 4.34E-06 | 3.05E-04 |
| GO:0015718 | monocarboxylic acid transport | BP | 5 | 1.31E-05 | 8.15E-04 |
| GO:0016042 | lipid catabolic process | BP | 6 | 2.11E-05 | 1.18E-03 |
| GO:0006644 | phospholipid metabolic process | BP | 6 | 5.15E-05 | 2.63E-03 |
| GO:0015711 | organic anion transport | BP | 6 | 1.96E-04 | 9.17E-03 |
| GO:0070071 | proton-transporting two-sector ATPase complex assembly | BP | 2 | 2.68E-04 | 1.16E-02 |
| GO:0046942 | carboxylic acid transport | BP | 5 | 3.61E-04 | 1.37E-02 |
| GO:0015849 | organic acid transport | BP | 5 | 3.66E-04 | 1.37E-02 |
| GO:0006869 | lipid transport | BP | 5 | 4.37E-04 | 1.53E-02 |
| GO:0010876 | lipid localization | BP | 5 | 6.80E-04 | 2.24E-02 |
| GO:0070374 | positive regulation of ERK1 and ERK2 cascade | BP | 4 | 9.92E-04 | 3.09E-02 |
| GO:1903038 | negative regulation of leukocyte cell-cell adhesion | BP | 3 | 1.89E-03 | 5.57E-02 |
| GO:0070528 | protein kinase C signaling | BP | 2 | 2.11E-03 | 5.92E-02 |
| GO:0047498 | calcium-dependent phospholipase A2 activity | MF | 5 | 2.66E-11 | 2.44E-09 |
| GO:0102567 | phospholipase A2 activity (consuming 1,2-dipalmitoylphosphatidylcholine) | MF | 5 | 8.97E-11 | 2.75E-09 |
| GO:0102568 | phospholipase A2 activity consuming 1,2-dioleoylphosphatidylethanolamine) | MF | 5 | 8.97E-11 | 2.75E-09 |
| GO:0004623 | phospholipase A2 activity | MF | 5 | 3.42E-09 | 7.86E-08 |
| GO:0004620 | phospholipase activity | MF | 5 | 6.49E-07 | 1.19E-05 |
| GO:0016298 | lipase activity | MF | 5 | 2.75E-06 | 4.22E-05 |
| GO:0052689 | carboxylic ester hydrolase activity | MF | 5 | 9.77E-06 | 1.28E-04 |
| GO:0005537 | mannose binding | MF | 3 | 1.34E-05 | 1.54E-04 |
| GO:0008201 | heparin binding | MF | 4 | 2.39E-04 | 2.45E-03 |
| GO:0048029 | monosaccharide binding | MF | 3 | 5.44E-04 | 5.00E-03 |
| GO:0005539 | glycosaminoglycan binding | MF | 4 | 7.07E-04 | 5.91E-03 |
| GO:0005543 | phospholipid binding | MF | 5 | 1.30E-03 | 9.73E-03 |
| GO:1901681 | sulfur compound binding | MF | 4 | 1.37E-03 | 9.73E-03 |
| GO:0042623 | ATPase activity, coupled | MF | 4 | 4.45E-03 | 2.92E-02 |
| GO:0016887 | ATPase activity | MF | 4 | 1.02E-02 | 6.25E-02 |
| GO:0008017 | microtubule binding | MF | 3 | 1.21E-02 | 6.96E-02 |
| rno00592 | alpha-Linolenic acid metabolism | KEGG | 5 | 9.72E-10 | 3.79E-08 |
| rno00591 | linoleic acid metabolism | KEGG | 5 | 1.52E-08 | 2.97E-07 |
| rno04975 | fat digestion and absorption | KEGG | 5 | 2.44E-08 | 2.97E-07 |
| rno00565 | ether lipid metabolism | KEGG | 5 | 3.05E-08 | 2.97E-07 |
| rno00590 | arachidonic acid metabolism | KEGG | 5 | 7.42E-07 | 5.79E-06 |
| rno00564 | glycerophospholipid metabolism | KEGG | 5 | 1.26E-06 | 8.16E-06 |
| rno04972 | pancreatic secretion | KEGG | 5 | 1.60E-06 | 8.91E-06 |
| rno04270 | vascular smooth muscle contraction | KEGG | 5 | 7.85E-06 | 3.83E-05 |
| rno04014 | ras signaling pathway | KEGG | 5 | 9.45E-05 | 4.09E-04 |
| rno04625 | C-type lectin receptor signaling pathway | KEGG | 3 | 1.52E-03 | 5.94E-03 |
| rno05162 | measles | KEGG | 3 | 3.36E-03 | 1.19E-02 |
| rno05152 | tuberculosis | KEGG | 3 | 6.16E-03 | 2.00E-02 |
| rno04145 | phagosome | KEGG | 3 | 7.39E-03 | 2.22E-02 |
