# Supplementary material for: Population genomics reveal rapid genetic differentiation in a recently invasive population of Rattus norvegicus
Source: Front Zool. 2021 Jan 26;18:6. doi: 10.1186/s12983-021-00387-z (PMC7836188; doi:10.1186/s12983-021-00387-z)
Supplement: Supplementary file 2 — Additional file 2: Figure S1. Tree topology inferred from TreeMix with m = 1 (a) and m = 2 (b). Group IDs correspond to those in Fig. 1. Figure S2. Detection of genetic mixture across all subgroups. Significance of 3 Population Test (Z) represents whether the corresponding subgroup (on Y axis) is of mixed ancestry of other subgroups. Each dot indicates the Z score of a test between the target subgroup and every pair of other subgroups. Positive value suggests a result of unadmixed. All the groups were showed with only positive values, suggesting a relatively unadmixed relationship to other subgroups. Group IDs correspond to those in Fig. 1. [file 12983_2021_387_MOESM2_ESM.docx]

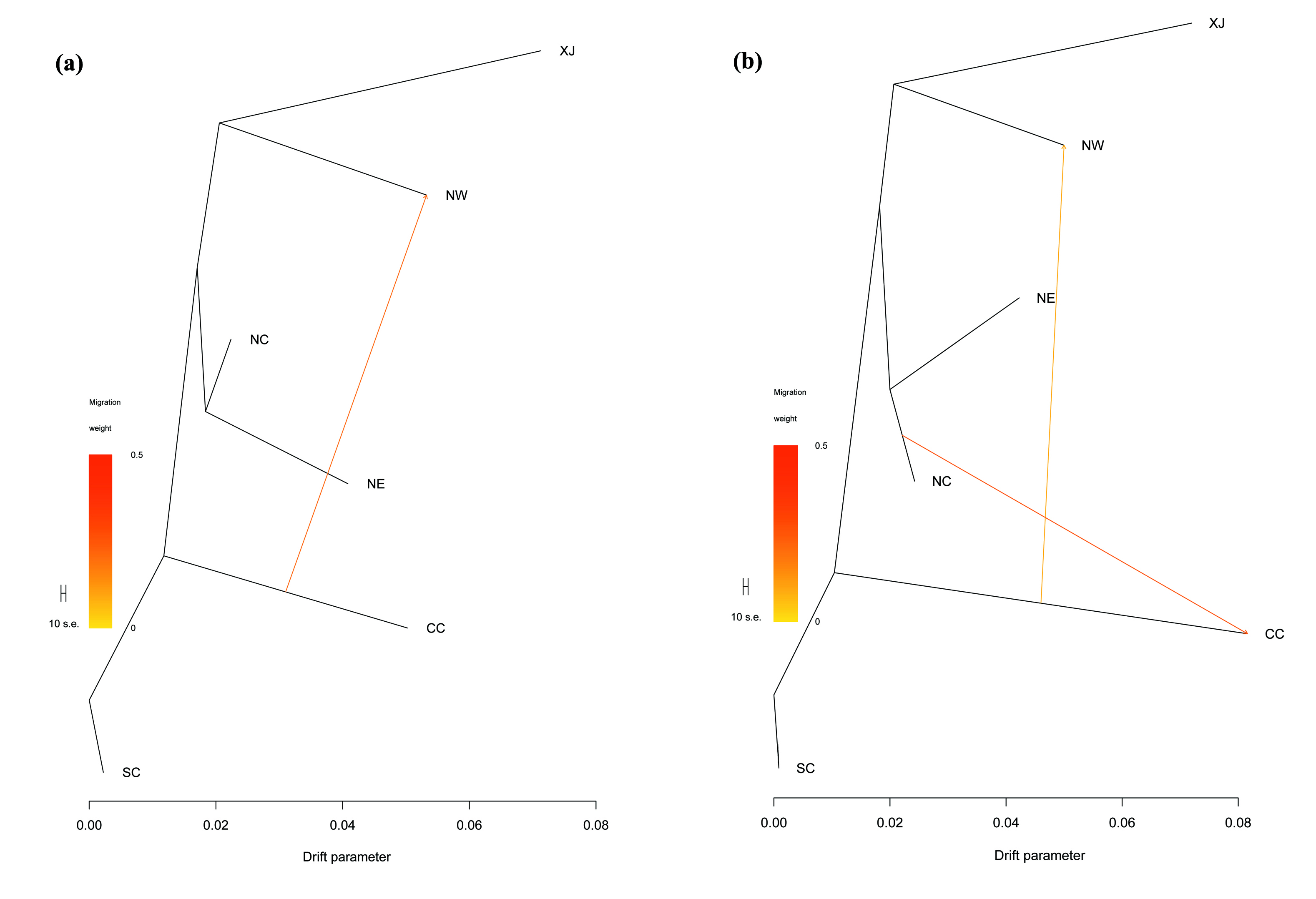


**Figure S1.** Tree topology inferred from TreeMix with m = 1 (a) and m = 2 (b). Group IDs correspond to those in Fig. 1.


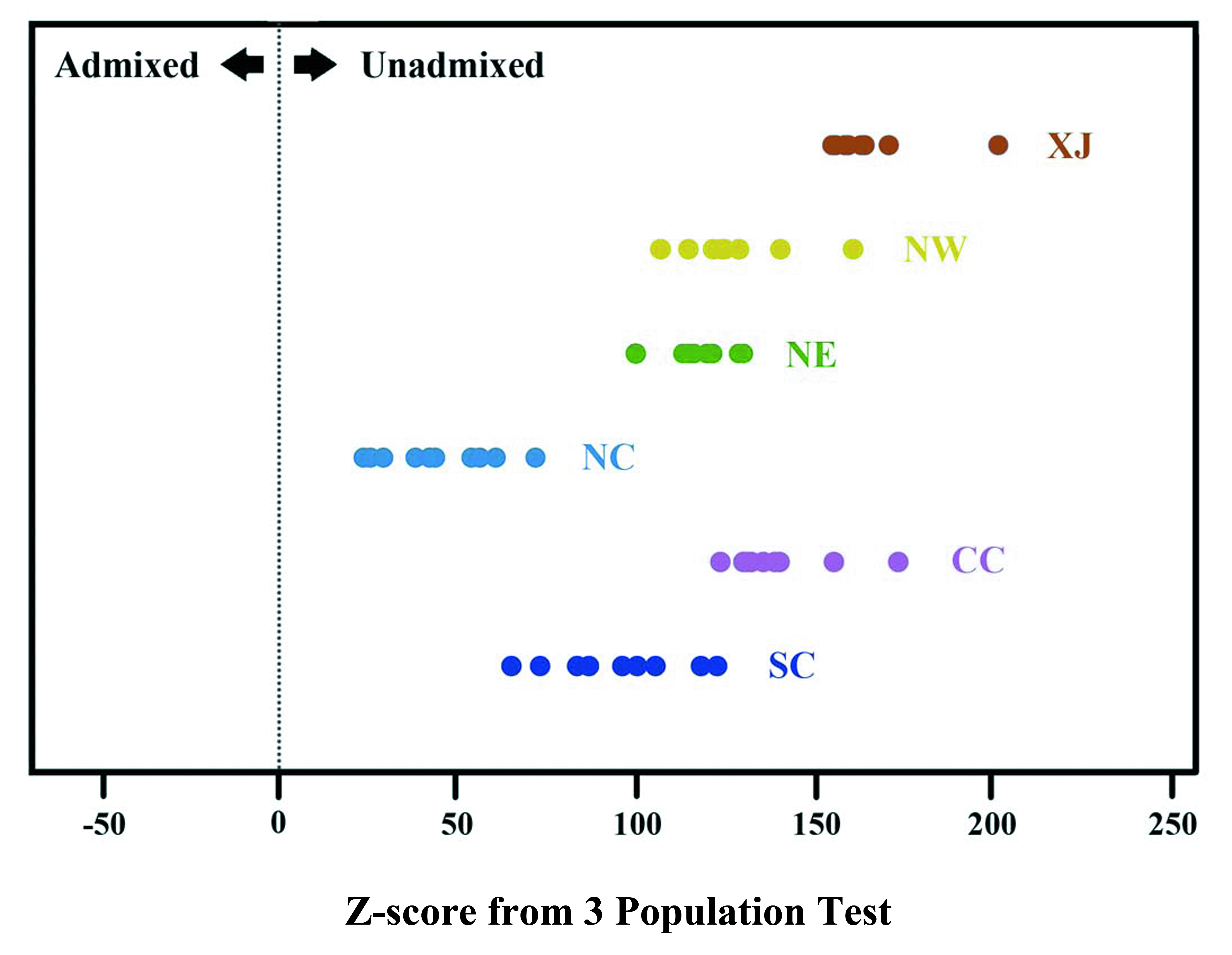


**Figure S2.** Detection of genetic mixture across all subgroups. Significance of 3 Population Test (Z) represents whether the corresponding subgroup (on Y axis) is of mixed ancestry of other subgroups. Each dot indicates the Z score of a test between the target subgroup and every pair of other subgroups. Positive value suggests a result of unadmixed. All the groups were showed with only positive values, suggesting a relatively unadmixed relationship to other subgroups. Group IDs correspond to those in Fig. 1.
